# Two-Step Cascade Process for Efficient Isolation of Cellulose from Sugarcane Trash

**DOI:** 10.3390/molecules31162860

**Published:** 2026-08-16

**Authors:** Jian Zhang, Jiang-Lin Yin, Liu-Yi Chen, Jie Gao, Bao-Yu Huang, Li-Ting Gao, Mei-Xia He, Xiang-Yi Li, Ri-Hui Lin, Da-Wei Li

**Affiliations:** 1 School of Chemistry and Chemical Engineering, Guangxi Key Laboratory for Polysaccharide Materials and Modification, Guangxi Minzu University, Nanning 530006, China; zhangj2015@gxtcmu.edu.cn (J.Z.); kuanggou731@163.com (L.-Y.C.); 17374878016@163.com (J.G.); 202313141100321@stu.gxmzu.edu.cn (B.-Y.H.); 202313141100505@stu.gxmzu.edu.cn (L.-T.G.); 202313141100441@stu.gxmzu.edu.cn (M.-X.H.); xiangyili00@163.com (X.-Y.L.); 2 Institute of Traditional Chinese and Zhuang-Yao Ethnic Medicine, Guangxi University of Chinese Medicine, Nanning 530200, China; yinjl@gxtcmu.edu.cn; 3 Key Laboratory of Eutrophication and Red Tide Prevention of Guangdong Higher Education Institutes, College of Life Science and Technology, Jinan University, Guangzhou 510632, China

**Keywords:** sugarcane trash, cellulose, green extraction, thermal stability, hydrogen bond

## Abstract

Efficient and sustainable isolation of cellulose from sugarcane tops (ST), a major sub-fraction of sugarcane trash, is of great significance for the high-value valorization of cellulose and its subsequent conversion into substrates for biomaterials. This study introduces a novel two-step cascade process under mild conditions, which integrates two treatments: a green multi-functional depolymerizing agent (MDA) treatment and the alkaline hydrogen peroxide (AHP) treatment. This sequential strategy is designed to selectively solubilize hemicellulose and lignin, respectively, ultimately yielding cellulose from ST. To assess extraction efficacy, four MDA treatment durations (0–3 h) were systematically investigated. The quality of the extracted cellulose was evaluated through lignocellulosic composition analysis, surface morphology, X-ray diffraction (XRD), Fourier-transform infrared spectroscopy (FTIR), and thermogravimetric analysis (TGA). Results demonstrate that the MDA treatment effectively disrupted ST’s recalcitrant structure, facilitating selective hemicellulose enrichment in deionized water and lignin dissolution in 1% (*w*/*v*) AHP. Notably, cellulose pretreated for 2 h exhibited preferred properties, including high purity (85.82%), a retention rate of 85.33%, preservation of the cellulose I crystalline polymorph (crystallinity index, *CrI* = 69.11%), and excellent thermal stability (maximum decomposition temperature, Tmax = 344.48 °C). The preserved structural integrity suggests the potential suitability of the extracted cellulose as a promising feedstock for polymer composite applications.

## 1. Introduction

Sugarcane trash, consisting of the leaves and tops discarded after harvest, accounts for 15–30% of total sugarcane biomass [1,2,3]. Although sugarcane trash is mainly used for low-value purposes such as feed or fuel [4], its high cellulose and hemicellulose content (≈70–80%) makes it a promising renewable feedstock for bioenergy production, including hydrogen [5], bioethanol [6,7,8], and other bio-based materials [9,10]. However, cellulose is tightly bound within a lignin–hemicellulose matrix (≈20–30%) [2,4], forming a recalcitrant structure which limits extraction and isolation of cellulose [11].

Conventional cellulose extraction methods always combined with techniques such as steam explosion [9,12], acid or alkaline treatment [10,13], and organosolv processes [14,15], extensively employed for cellulose isolation from sugarcane trash, often result in considerable cellulose mass loss (25–35%), leading to insufficient overall yields (<70%). Hence, an efficient extraction process is required to improve cellulose accessibility and yield.

Moreover, systematic studies on the preservation of sugarcane trash cellulose structure and the evolution of its physicochemical properties remain limited. An optimized alkali-H_2_O_2_ bleaching process increased cellulose purity by 41%, crystallinity by 20%, and onset thermal decomposition temperature from 256.97 °C to 317.78 °C [10]. Bleaching with hydrogen peroxide significantly increased the viscosity of cellulose, which, in turn, promoted its suitability for downstream derivative synthesis [13]. It should be noted, however, that bleaching conditions must be optimized for each type of lignocellulosic biomass, as the efficiency of hydrogen peroxide delignification is highly dependent on the specific chemical composition and structural characteristics of the feedstock [16]. Due to these limited characterizations, the physicochemical evolution of sugarcane trash cellulose remains unclear. Hence, there is a need to make a clear structure–property synergy of cellulose derived from biomass in order to clarify its application potential.

Most previous studies have treated sugarcane trash as a bulk feedstock without specifying the proportions of its senescent leaves, green leaves, and tops, which vary considerably with harvesting practices. This lack of material specificity inevitably confounds the correlation between extraction parameters and cellulose properties, limiting the establishment of reliable structure–property relationships. In this study, we specifically target sugarcane tops (ST), the apical growing stem portion with tender leaves, as a well-defined sub-fraction of sugarcane trash. Compared with senescent leaves, tops are physiologically younger and possess a lignocellulosic matrix with lower lignin content and altered structure, rendering it more susceptible to structural rearrangement during chemical fractionation. By narrowing the feedstock scope to tops, we reduce the inherent variability associated with bulk sugarcane trash and enable a clearer understanding of how specific biomass structures affect cellulose extraction efficiency and quality.

The objective of this study is to develop a green and efficient cascade process for the isolation of high-purity cellulose from sugarcane tops through the progressive removal of hemicellulose and lignin and to systematically characterize the structural and physicochemical evolution of the resulting cellulose throughout the process. Unlike conventional liquid-phase pretreatments that consume large volumes of reagents, this process features an MDA pretreatment conducted under an ultralow liquid-to-solid ratio, where proton-donating species vaporize and diffuse into the biomass matrix to selectively cleave intercomponent linkages. The findings are expected to provide mechanistic insights into cellulose structural evolution and to establish a foundation for linking process parameters to cellulose quality, thereby informing the rational design of biomass fractionation strategies.

## 2. Results

### 2.1. MDA Treatment Alters the Cell Wall Structure of ST

**Figure 1:**
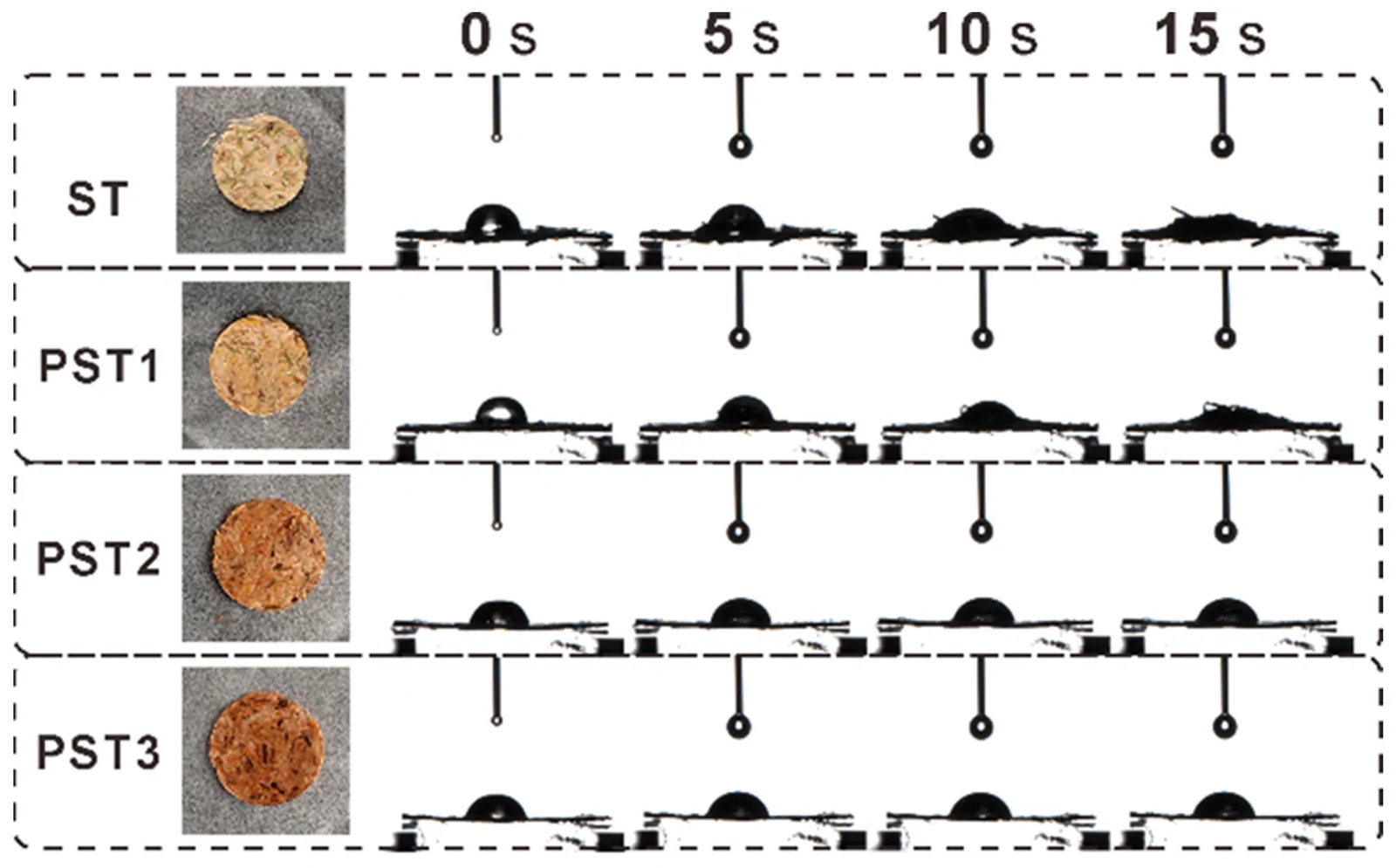
Contact angle test of ST and PST1-3 tablets.

**Figure 2:**
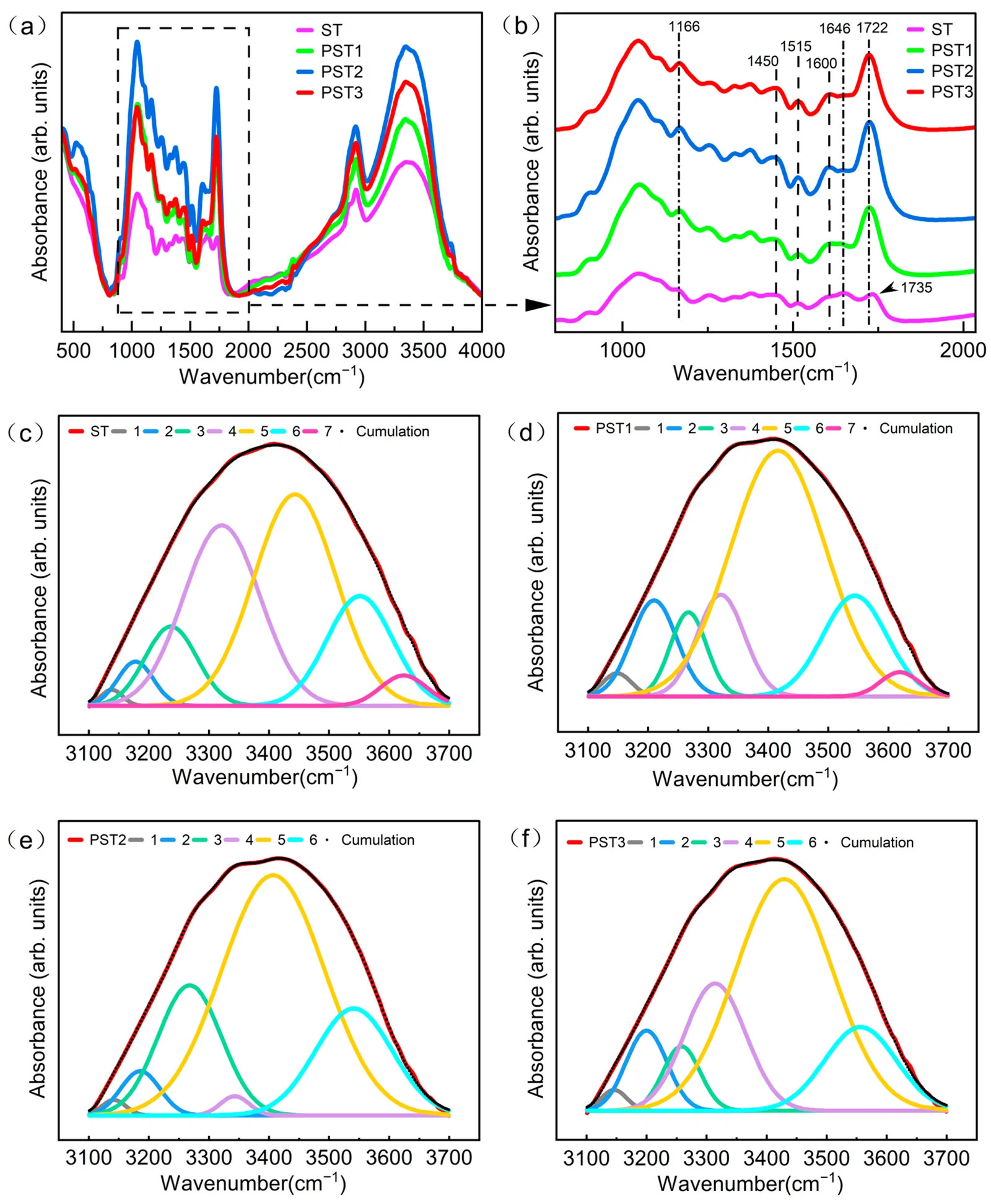
The FTIR absorbance spectra of ST and PST samples (**a**), the enlarged view of 800 to 2000 cm^−1^ (**b**) and the peak separation of hydrogen bonding OH stretching from 3100 to 3700 cm^−1^ (**c**–**f**).

Due to the extremely low material-to-liquid ratio (1/1.6, wt./v), MDA treatment markedly altered ST’s color, without generating a separate liquid phase during the MDA treatment. This avoids solid–liquid separation during the MDA reaction itself; however, the subsequent washing and AHP stages generate liquid streams whose impacts require quantitative assessment. To assess the influence of MDA treatment on cell wall disassembly, four different treatment durations (1, 2 and 3 h) were applied, and the resulting products were named PST1, PST2 and PST3 respectively (PST was MDA pretreated sugarcane tops; the numbers refer to the treatment duration). Contact angle measurement and FTIR spectroscopy were used, and the results are shown in Figure 1 and Figure 2.

As Figure 1 shows, the raw ST tablet fully disintegrated within 15 s of water-droplet contact due to its high porosity and loose structure. In contrast, the pretreated tablet PST1 absorbed water slowly while maintaining structural integrity. Notably, droplets on PST2 and PST3 tablets remained upright without deformation. These observations confirm that MDA treatment increases the compressibility and compactness of sugarcane trash materials. The distinct water penetration and stability differences reflect fundamental internal structural changes: compared to ST’s high-porosity, well-connected pores, PSTs exhibit reduced porosity, impaired pore connectivity, and stronger interparticle bonding, enabling them to resist water-induced disintegration.

Compared with ST, PSTs exhibited markedly stronger characteristic peaks, with PST2 showing the highest overall intensity, indicating the most extensive exposure of surface functional groups (Figure 2a). In the enlarged 800–2000 cm^−1^ region (Figure 2b), the bands attributed to the lignin aromatic ring skeleton (1600 and 1510 cm^−1^) and C–H vibration (1450 cm^−1^) [11,17], as well as the C–O stretching of hemicellulose and cellulose (1166 cm^−1^) [11,18,19], were all enhanced. The C=O stretching band shifted from 1735 to 1722 cm^−1^ and showed a pronounced increase in intensity, reflecting the degradation of hemicellulose and lignin along with partial esterification [18,20]. In addition, the faint 1646 cm^−1^ band observed in PST2 and PST3 indicated a reduction in adsorbed water.

The -OH absorption band near 3400 cm^−1^ strengthened substantially, suggesting that structural deconstruction exposed additional hydroxyl groups and reconfigured the hydrogen-bond network linking cellulose, hemicellulose, and lignin within the plant cell wall [21,22,23,24,25,26]. Deconvolution of the 3100–3700 cm^−1^ region (Figure 2c–f; Table 1) revealed that intermolecular hydroxyl groups (inter -OH, i.e., hydrogen bonds formed between the three major components, including Peaks 1–4) content declined from 43.30% (ST) to 24.92% (PST2) before rising slightly to 33.03% (PST3), while intramolecular hydroxyl group contents (intra -OH, i.e., hydrogen bonds within individual molecules of each of these three components, represented by Peak5) first increased from 37.97% to 56.46% before declining to 52.97%. Overall, MDA treatment reduced inter -OH and strengthened intra -OH within the supramolecular framework, with PST2 exhibiting the most pronounced transition. Regarding free -OH groups (represented by peak6 and 7), both ST and PST1 retained two types, whereas PST2 and PST3 contained only the low-wavenumber form; among the treated samples, PST2 showed the highest relative abundance. Across the ST and PST samples, inter-OH components occurred at 3138–3343 cm^−1^ (Peaks 1–4), intra -OH at 3407–3443 cm^−1^ (Peak 5), and free -OH at 3541–3624 cm^−1^ (Peaks 6–7); the high-wavenumber free -OH component near 3619–3624 cm^−1^ was not detected in PST2 or PST3.

These findings suggest that proton donors in MDA generate H_3_O^+^, which selectively disrupts the hydrogen-bond networks linking cell wall components [27]. Concurrently, esterification reactions between MDA and surface hydroxyl (-OH) groups consume part of these hydroxyls and introduce steric hindrance, which promotes intramolecular hydrogen-bond formation, induces structural rearrangement, and substantially increases the exposure of functional groups. As shown in the microscopic observations of lignin-stained samples (Figure S1), a noticeable red color was observed in PST2, whereas only a weak red signal was detected in ST. This finding suggests that lignin in PST2 was significantly more liberated and accessible. The intrinsic properties of lignin, that is, its weak hydrogen bonding and strong hydrophobicity [24,28,29], approximately affect its solubility. Overall, the MDA treatment effectively breaks the recalcitrant structural barrier of ST, providing a solid basis for subsequent component fractionation.

### 2.2. MDA Treatment Promoted the Water Solubility of Hemicellulose

**Figure 3:**
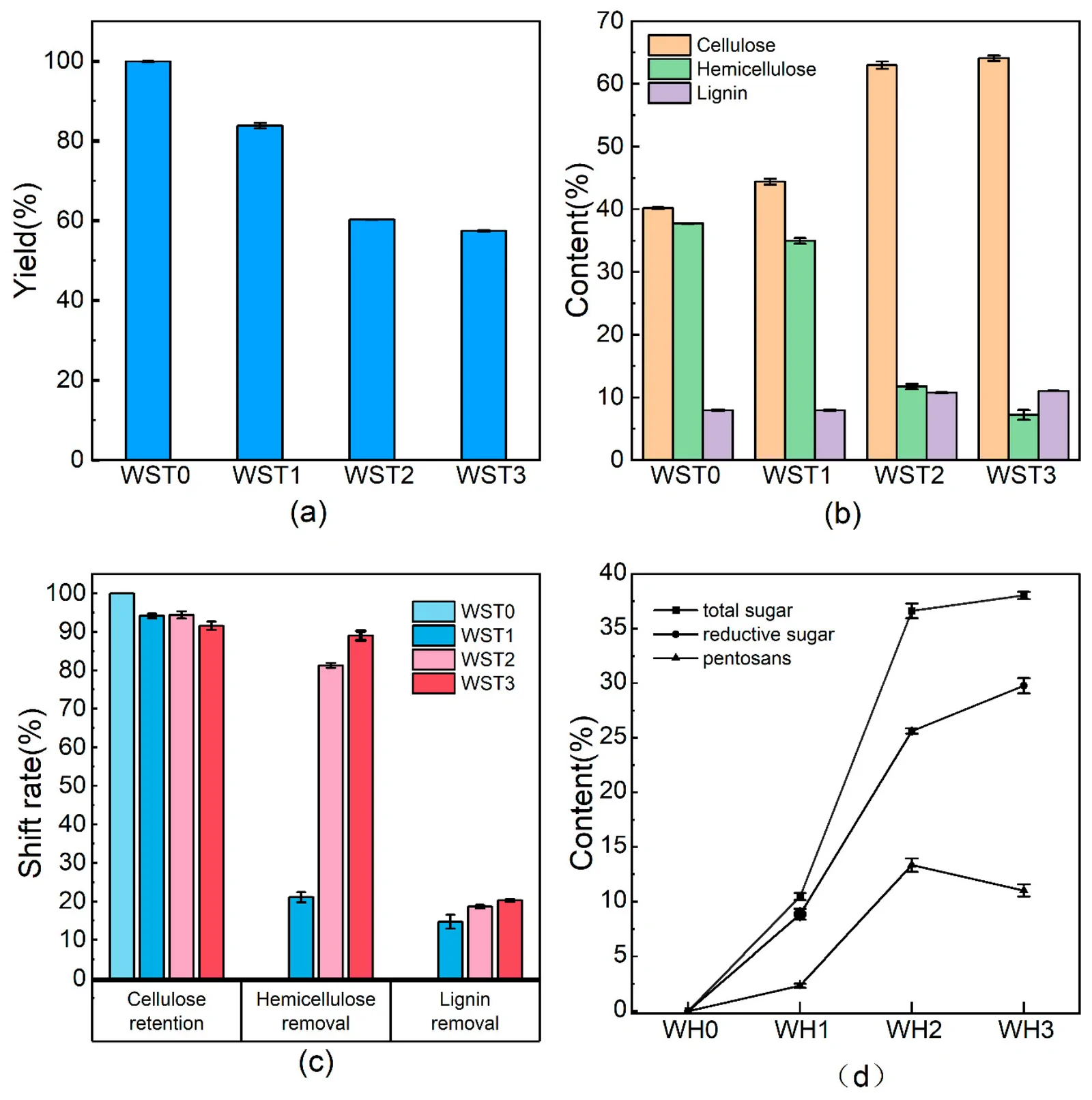
Yield (**a**), chemical component contents (**b**) and shift rates (**c**) of the solid residuals and sugar contents in water filtrate portions (**d**) for MDA treatment.

With the extension of MDA treatment duration, the mass of residual solids (WST, washed solid residue) decreased from approximately 100% to 57.42% (Figure 3a). Figure 3b presents the analysis results of the main chemical components of the solid residues, where the composition of WST0 is consistent with that of ST. From WST0 to WST3, the cellulose content obviously increased from 40.19% to 64.10%, with a percentage increase of 59.49%; the lignin content rose from 7.95% to 11.04%, corresponding to a 38.87% increase; in contrast, the hemicellulose content declined markedly from 37.75% to 7.20%, showing a remarkable decrease rate of 80.92%. The specific percentage changes in these three main components compared with the original raw material (ST) are illustrated in Figure 3c. The results indicated that under MDA treatment for 1–3 h, the cellulose loss was less than 10% in all cases, and the lignin removal rate ranged from 14.70% to 20.26%. Notably, the hemicellulose removal rate sharply increased from 21.08% (1 h) to 81.26% (2 h) and slightly rose to 89.03% at 3 h. These findings demonstrate that MDA treatment exhibits a strong separation effect on hemicellulose while effectively retaining cellulose, with the highest treatment efficiency achieved at 2 h.

Furthermore, the sugar content in the aqueous solution was analyzed to clarify the effect of MDA on the dissolution behavior of hemicellulose. As shown in Figure 3d, from WH0 to WH3 (WH, water filtrate), the contents of total sugar and reducing sugar exhibited an overall upward trend, with reducing sugar accounting for 70% to 80% of the total sugar. The pentose content first increased and then decreased, reaching its maximum value (13.35%) in WH2, and this trend may be attributed to the intensified oxidative decomposition of xylose after 2 h of treatment. Particularly, when the treatment duration was extended from 2 h to 3 h, the increase in the amplitude of reducing sugar (4.17%) was higher than that of total sugar (1.43%). This suggests that prolonging the MDA treatment duration not only promotes the further dissolution of hemicellulose components but also induces the cleavage of more glycosidic bonds, accompanied by the further oxidative decomposition of xylose. The WH filtrates were characterized only by aggregate total sugar, reducing sugar, and pentose measurements; individual sugars, fermentation inhibitors, and purification requirements were not determined. Because dissolved non-sugar components and handling losses during filtration, washing, and drying were not quantified, the reported solid yields and component shift rates do not constitute a complete stage-wise process mass balance.

Consistent with the above results, MDA treatment improved markedly the water solubility of ST. It is speculated that MDA treatment can effectively hydrolyze glycosidic bonds in the amorphous regions, which is further supported by the increased reducing sugar content observed in the aqueous phase (Figure 3d), thereby leading to the preferential dissolution of hemicellulose, which is an important structural polysaccharide in cell walls. This is consistent with the high hemicellulose removal rate (up to 81.26% at 2 h) demonstrated earlier. Due to the loss of hemicellulose, which acts as a cross-linking agent in the cell walls of sugarcane top shoots, the internal structure collapsed, resulting in a shrunken and wrinkled micromorphology (Figures S2 and S3).

### 2.3. Accelerated Cellulose Extraction via Lignin Dissolution in 1% AHP: Dependent on MDA Treatment Duration

**Figure 4:**
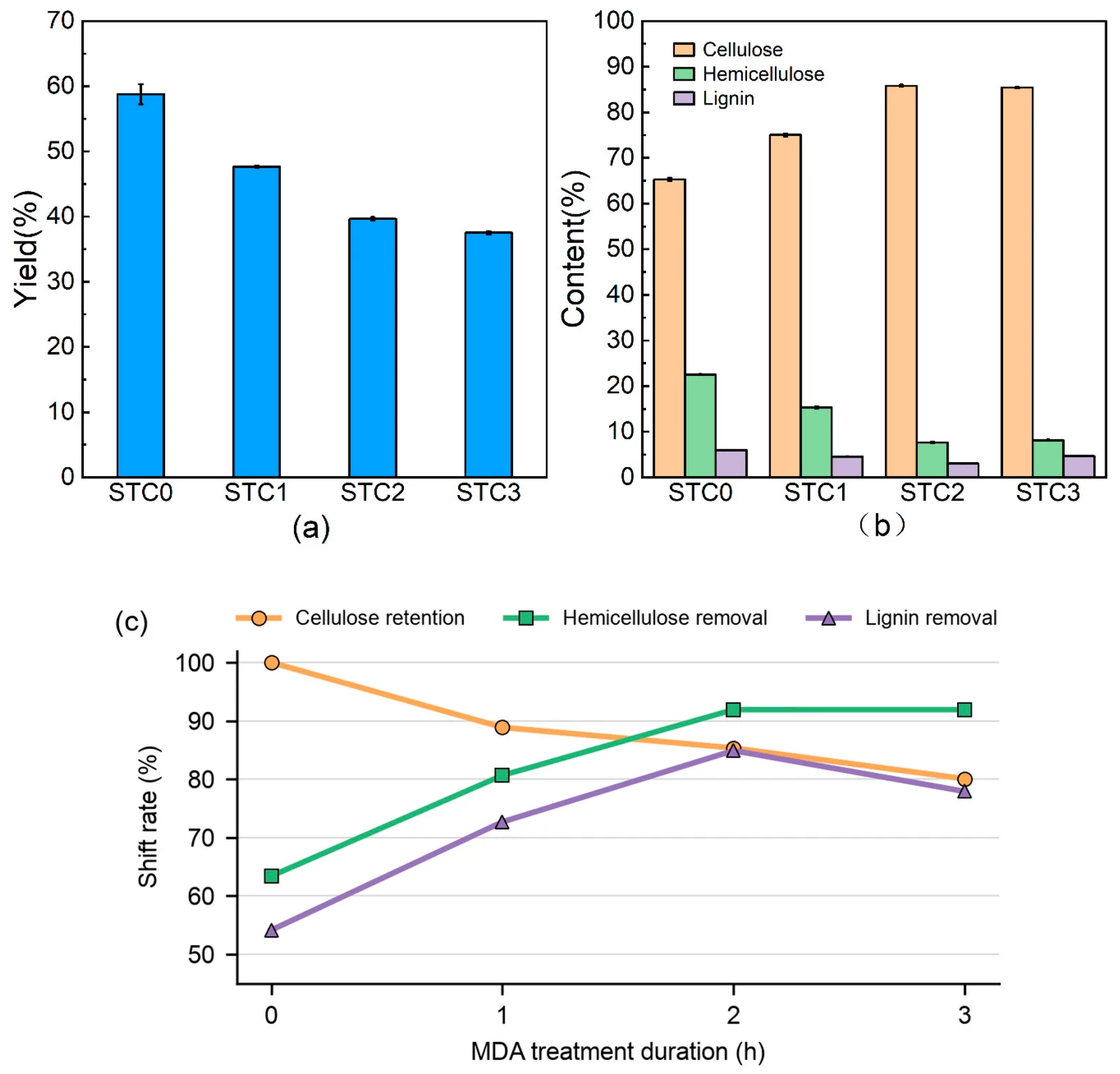
Yield (**a**), chemical component contents (**b**) and shift rates (**c**) of solid samples for AHP treatment. In panel (**c**), lines connect the mean values across MDA treatment durations (0–3 h).

Figure 4a shows that treatment with 1% AHP further reduced the solid residual mass, with a decreasing trend consistent with that observed in the MDA treatment stage; specifically, the longer the MDA treatment duration, the lower the residual mass (declining from 58.79% to 37.53%). The key difference was that, besides facilitating the further removal of residual hemicellulose during this stage, the primary efficacy of MDA treatment lay in promoting lignin dissolution in 1% AHP. This was evidenced by Figure 4b,c: the cellulose content increased markedly, with that of STC2 (STC, sugarcane tops cellulose) reaching 85.82% (Figure 4b) and the corresponding cellulose retention rate as high as 85.33% (Figure 4c). Compared with the hemicellulose removal rate of STC0 (63.40%), hemicellulose removal peaked after 2–3 h of MDA treatment, with the removal rate exceeding 90%. Notably, lignin was predominantly solubilized during the 1% AHP stage, and this process was accelerated by the preceding MDA treatment.

In contrast to MDA-untreated STC0, which exhibited a lignin removal rate of only 54.12% during the AHP stage, all MDA-treated samples achieved cumulative lignin removal rates above 70%, with STC2 showing the highest value (up to 84.89%). A comparative analysis combining Figure 3c and Figure 4c revealed the lignin removal rates in the sole AHP stage: 57.97% for STC1, 66.27% for STC2 and 57.62% for STC3, representing respective increases of 7.11%, 22.46% and 6.47% relative to this value recorded for STC0. These results demonstrated that 2 h of MDA treatment was most favorable for subsequent lignin dissolution during the subsequent AHP treatment. Specifically, 1 h of treatment led to insufficient cell wall depolymerization, whereas 3 h of treatment likely triggered lignin condensation [27,30]. This condensation rendered lignin difficult to dissolve in the low concentration of 1% AHP, which would presumably require a higher AHP concentration to achieve effective dissolution; however, an excessively high AHP concentration would in turn pose a considerable risk of cellulose loss. Thus, selecting the 2 h MDA treatment duration achieves a desirable balance between efficient lignin removal and cellulose preservation, resolving the inherent trade-off in low-concentration AHP-based two-step treatment processes. This finding provides critical insights for the development of cost-effective and cellulose-friendly biomass fractionation technologies involving sequential MDA and AHP treatments.

### 2.4. Characterizations of Cellulose Extracts

**Figure 5:**
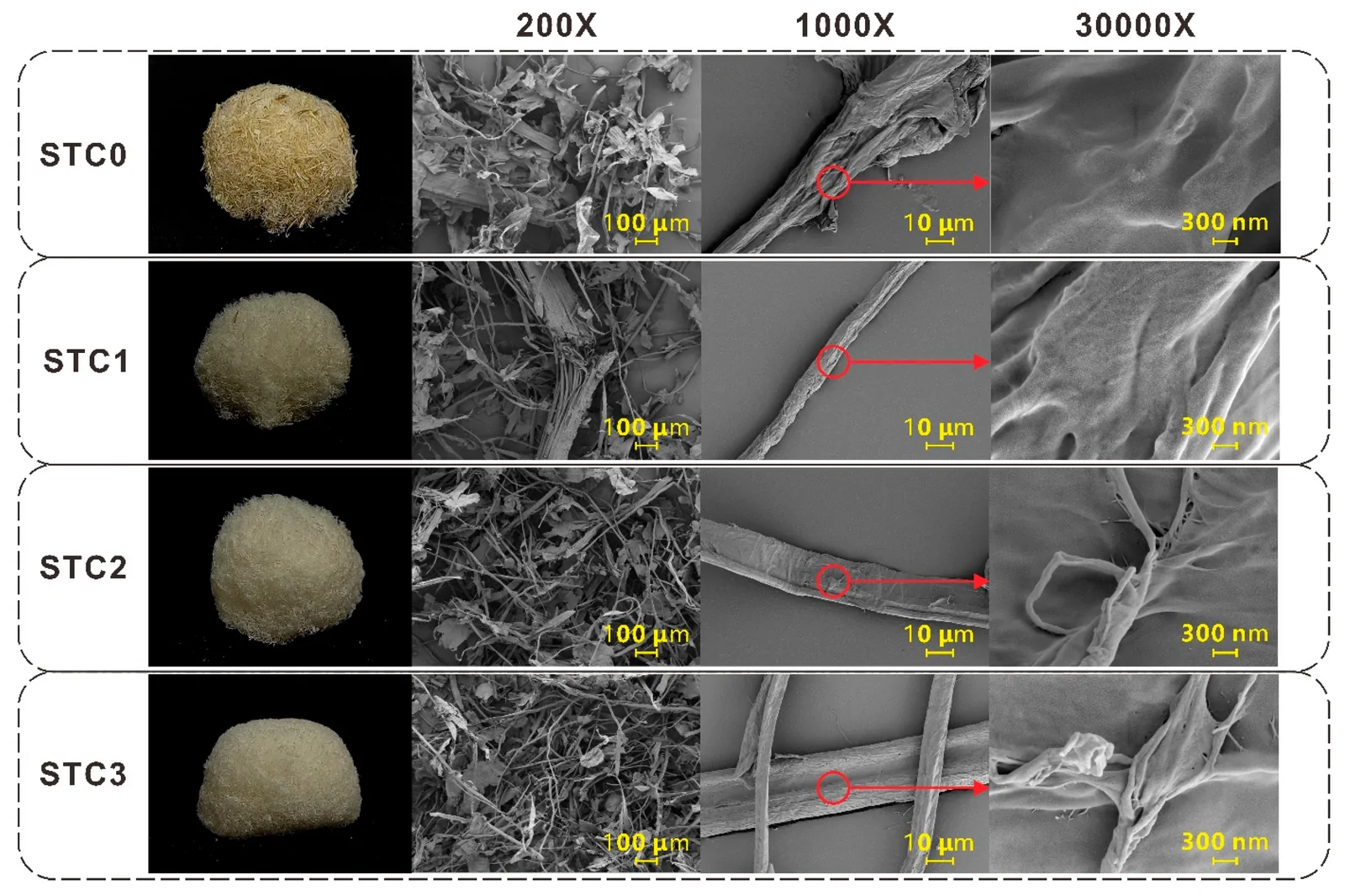
Digital photo and FE-SEM micrograph of cellulose samples.

Comprehensive morphological, structural, and thermal analyses revealed that solvent-free pretreatment profoundly altered the physicochemical properties of sugarcane trash cellulose (STC0–STC3). As shown in Figure 5, the untreated STC0 appeared dark yellow and heterogeneous, while STC1 was slightly yellowish, and STC2 and STC3 were white, fibrous, and residue-free. FE-SEM images (200×) showed that STC0 and STC1 retained vascular bundles up to 100 µm in diameter, whereas STC2 and STC3 consisted mainly of individual fibers 5–20 µm wide, consistent with previous reports on delignified ST fibers [9,12]. The transition from compact vascular bundles to individual fibers indicates that the extended pretreatment effectively removed the cementing matrix (hemicellulose and lignin) from the middle lamella and cell corners. Higher-magnification images (1000×–30,000×) revealed smooth, finely wrinkled micro-surfaces similar to those of alkaline-treated fibers [31], with distinct microfibrils clearly visible in STC2 and STC3. This suggests that extending the pretreatment beyond 2 h promotes the removal of hemicellulose and lignin that cross-link microfibrils. Collectively, the foregoing chemical composition measurements show that STC2 and STC3 have similar total removal efficiencies of hemicellulose and lignin and comparable cellulose purity, consistent with the microscopic morphology results in this section.

**Figure 6:**
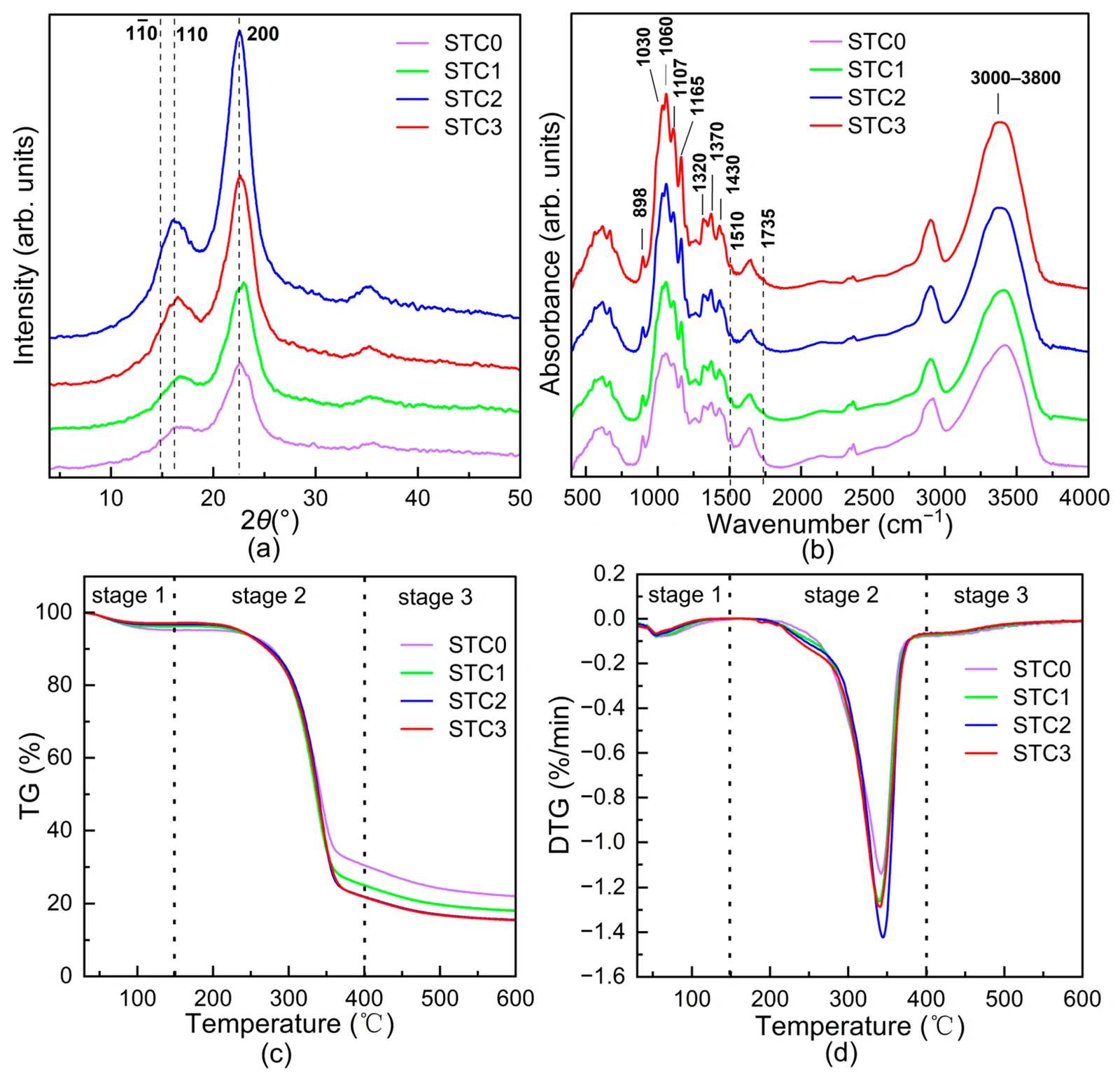
(**a**) X-ray diffractograms, (**b**) FTIR spectra, (**c**) TG curves and (**d**) DTG curves of extracted cellulose samples.

XRD patterns displayed the characteristic peaks of cellulose I at 16.5°, 22.5°, and 35°, corresponding to the (110), (200), and (004) planes (Figure 6a) [18]. Among all samples, STC2 exhibited the highest crystallinity index (69.11%), followed by STC3 (67.35%), indicating that excessive pretreatment partially disrupted the crystalline regions and converted them into amorphous structures [32]. FTIR spectra further supported this trend: cellulose-related bands at 898, 1107, and 1165 cm^−1^ (β-glycosidic linkages), 1060 and 1030 cm^−1^ (C-O stretching of the pyranose ring), and 1320–1430 cm^−1^ (CH_2_ bending) intensified with increasing pretreatment time (Figure 6b), while lignin (1510 cm^−1^) and hemicellulose (1735 cm^−1^) peaks gradually weakened. These changes confirm that solvent-free pretreatment efficiently enriched cellulose while maintaining its native structural integrity.

Deconvolution of the hydroxyl-stretching region (3000–3800 cm^−1^; Table 2, Figure S4 and Table S1) revealed a marked shift in hydrogen-bonding patterns. STC0 and STC1, resembling cellulose extracted under ambient conditions [33], were dominated by intra -OH (i.e., intrachain hydrogen bonds within individual cellulose chains, with characteristic peaks at around 3350 and 3470 cm^−1^), that is, 72.53% and 55.95%, respectively. In STC3, the intra -OH content decreased to approximately 47%, matching the inter -OH (i.e., interchain hydrogen bonds between adjacent cellulose chains) level, while STC2 showed an inter -OH/intra -OH ratio of 1.38, indicating strengthened intermolecular hydrogen bonding and enhanced thermal stability [17,34,35]. In the STC series, the inter -OH components shifted from 3116 and 3219 cm^−1^ in STC0 to 3136 and 3302 cm^−1^ in STC2, whereas the intra-OH components occurred near 3350 and 3443–3473 cm^−1^; the corresponding relative areas are reported in Table S1.

Thermogravimetric analysis showed three distinct stages: moisture evaporation (room temperature–150 °C), carbohydrate decomposition (150–400 °C), and carbonization (400–600 °C) (Figure 6c,d). During the main degradation phase, hemicellulose, cellulose, and lignin decomposed sequentially, with lignin exhibiting a broader degradation range extending beyond 400 °C [19,36,37]. With increasing pretreatment intensity (STC0 to STC3), the residue at 400 °C decreased from 30.43% to approximately 22%, reflecting higher cellulose purity. The maximum degradation temperature (T_max_) ranged from 340 to 345 °C, with STC2 exhibiting the highest value (344.48 °C), comparable to that of other biomass celluloses [33,38]. The superior purity, crystallinity, and strong inter -OH bonding of STC2 [17,39] collectively conferred the best thermal stability, surpassing that of STC0, whose apparent resistance primarily resulted from impurity-related degradation barriers [36]. These results indicate that STC2 cellulose possesses preferred structural and thermal properties that may support future evaluation in composites and textiles.

## 3. Discussion

In recent years, major sugarcane-producing countries such as Brazil and Thailand have focused on the valorization of the abundant biomass residues generated after harvest, commonly referred to as sugarcane trash, which includes tops, leaves, and other field residues. Particular attention has been paid to the extraction of cellulose and its conversion into cellulose nanocrystals [9,10,14].

As shown in Table 3, conventional methods such as steam explosion, acid-alkali treatments, and bleaching remain the dominant strategies for cellulose extraction from sugarcane trash. These approaches generally suffer from high energy consumption, environmental concerns, and a notably high cellulose loss rate ranging from 25% to 63%. Beyond these issues, a recurring challenge lies in the inherent trade-off between cellulose recovery and purity, where optimizing one parameter invariably comes at the cost of the other. For instance, one study reported a cellulose purity of 85% but with a recovery rate of only 37.02% [9], whereas another achieved a recovery rate of 74.13% while failing to reach 85% purity [13]. In contrast, the gas–liquid–solid multiphase reaction with MDA employed in this study achieves a better balance, yielding a cellulose recovery rate of 85.33% with a purity of 85.82%. This dual advantage suggests clear potential for energy savings, emission reduction, and improved economic viability, which requires further pilot-scale validation.

It should be noted that direct comparisons across the studies in Table 3 are constrained by differences in feedstock definition, pretreatment severity, and calculation methodologies. Most cited studies employ “sugarcane trash” as a collective term without distinguishing between senescent leaves, green leaves, and tops. These fractions differ substantially in composition and properties. Consequently, variations in cellulose purity and recovery may arise not only from processing conditions but also from feedstock heterogeneity, a variable largely uncontrolled in previous reports. By narrowing our feedstock to a well-defined sub-fraction, specifically sugarcane tops, we minimize this variability and enable a more reliable correlation between processing parameters and product quality. This approach underscores the importance of material definition in cross-study comparisons. With this feedstock-specific foundation established, we can now more critically examine the mechanisms underlying cellulose loss during conventional extraction processes.

The mechanistic interpretations in the following paragraphs are hypotheses inferred from compositional and spectroscopic evidence rather than direct observations of reaction intermediates. It is hypothesized that conventional extraction methods lead to cellulose loss by exposing cellulose through excessively solubilizing hemicellulose and lignin, thereby making it more vulnerable to degradation under alkaline conditions [13] or by hydroxyl radicals generated from hydrogen peroxide [14]. In a related study [40], two different bleaching agents were applied to treat the alkaline-pretreated durian husk (wherein most hemicellulose had been removed). The results showed that sodium hypochlorite (NaClO) achieved higher delignification efficiency compared to the combined bleaching system of sodium chloride (NaCl) and acetic acid (CH_3_COOH). However, cellulose yield decreased by 21.54%, a loss attributed to the strong oxidizing activity of NaClO.

While these explanations are plausible, they largely overlook the interdependence between successive extraction steps. In particular, the first treatment step likely alters the supramolecular structure of lignocellulose, which in turn affects the efficiency of subsequent cellulose extraction. Our study takes this aspect into account. The MDA treatment appears to mitigate this issue by promoting hydrogen bond rearrangement among cell wall components while strengthening intramolecular hydrogen bonds within cellulose, thereby enhancing its stability during the subsequent AHP treatment.

Moreover, the present process demonstrates markedly higher hemicellulose separation efficiency, solubilizing 81.25% in water after 2 h of MDA treatment while generating a sugar-containing aqueous filtrate. The resulting sugar-rich filtrate may have fermentation potential, including for single-cell protein production, but this use requires additional characterization and validation, which is currently under investigation in our lab. Compared with the conventional hydrothermal method [41,42], this approach more effectively cleaves hydrogen bonds and glycosidic bonds associated with hemicellulose. Unlike high-concentration acid or alkali treatments [31,43,44], which typically sacrifice the recoverability and quality of hemicellulose, the strategy preserves its utility for downstream applications.

It should also be noted that lignin was effectively extracted in the second step using only 1% AHP (1% H_2_O_2_ + 1% NaOH), achieving a removal rate of 84.89%. This performance surpasses that of conventional high-concentration AHP systems [31,43,45,46]. It is hypothesized that the MDA pretreatment may weaken intermolecular interactions, promote the cleavage of LCC linkages and lower the energy barrier for ·OOH radical attack on the Cα-Cβ bonds of lignin phenylpropane units [47]. Consequently, efficient lignin separation is achieved with lower chemical demand; its comparative environmental performance remains to be quantified.

**Figure 7:**
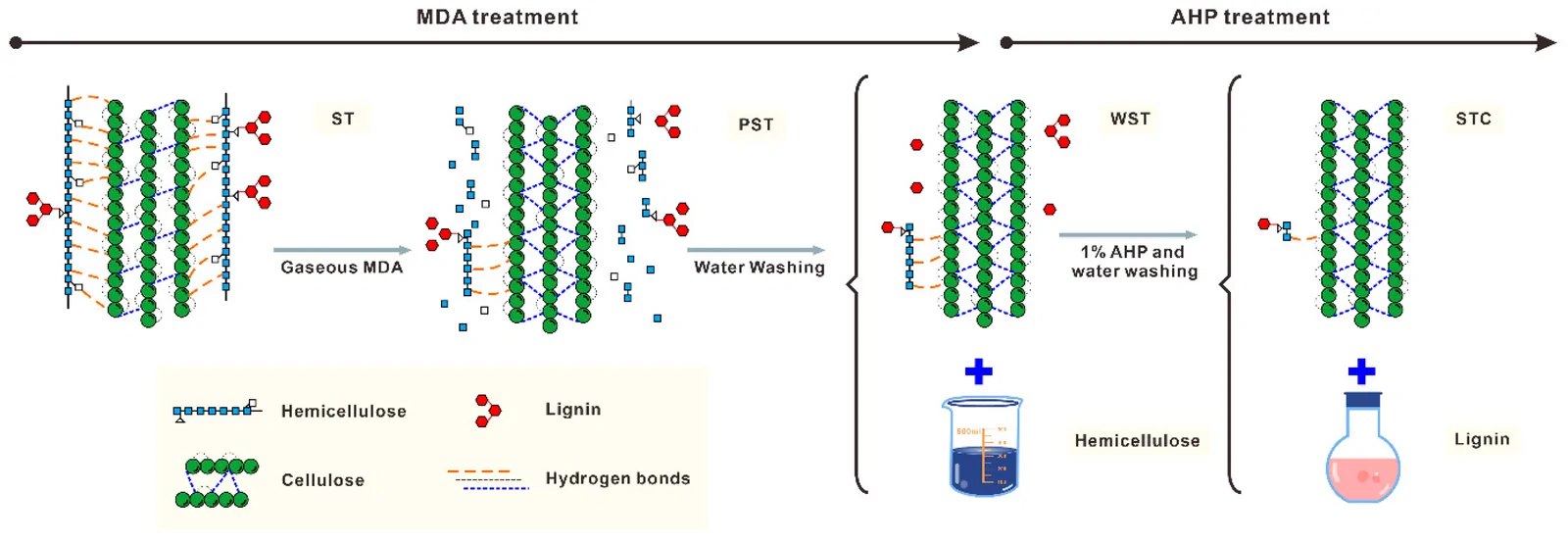
The proposed mechanism schematic diagram of the sequential MDA-AHP process.

Based on the preceding analysis, a proposed mechanism for component separation by the sequential MDA-AHP process is illustrated in Figure 7. The schematic depicts a hypothesized pathway in which MDA treatment, via a gas–liquid–solid multiphase reaction, preferentially disrupts key intermolecular linkages, including hydrogen bonds, hemicellulose glycosidic bonds, and lignin–carbohydrate complexes. This targeted disruption is proposed to facilitate the subsequent dissolution of hemicellulose in water and lignin in the dilute AHP solution, leading to a purified cellulose-rich solid residue.

In summary, the novelty of this work lies in two aspects. First, from a research content perspective, most previous studies have treated sugarcane trash as a bulk feedstock and focused primarily on maximizing yield or purity, whereas systematic investigations into the structural preservation and physicochemical evolution of the extracted cellulose remain limited. Our study addresses this gap by tracking the structural changes throughout the extraction process, providing a clearer understanding of the structure–property synergy of cellulose derived from biomass. Second, from a technological principle perspective, the MDA pretreatment operates at an ultralow liquid-to-solid ratio (1.6 mL/g) under a gas–liquid–solid multiphase system, where proton-donating species vaporize and diffuse into the cell wall matrix, where they are proposed to selectively cleave glycosidic bonds and weaken hydrogen-bond networks. This vapor-phase proton transfer mechanism minimizes liquid waste and avoids excessive cellulose degradation. Collectively, these features establish the MDA-AHP cascade as a promising and sustainable strategy for sugarcane trash valorization.

## 4. Materials and Methods

### 4.1. Materials

Raw sugarcane tops (ST), the apical growing stem portion with tender leaves and a sub-fraction of sugarcane trash, were collected from Nanning, Guangxi Province, China. Before being further screened to granules with sizes between 18 and 40 mesh (0.43 to 0.85 mm), the samples were squeezed to extract juice, washed to remove the soluble sugar, and air-dried. The chemical composition (wt.%, oven-dry-weight basis) analysis showed that ST contained 40.19 ± 0.13% cellulose, 37.75 ± 0.07% hemicellulose, and 7.95 ± 0.13% lignin, according to the Van Soest method [48]. MDA is an aqueous acidic agent with proton-donating capability, provided by the Team for the Development and Application of Agricultural and Forestry By-Products, Guangxi Minzu University. The stock solution has a pH of 1.8–2.5 and a boiling point of approximately 105 °C. Prior to use, the stock solution was diluted with deionized water at a volume ratio of 3:5 to prepare the working solution. Sodium hydroxide (NaOH) and hydrogen peroxide (H_2_O_2_, 30%) (Sinopharm Chemical Reagent Co., Ltd., Shanghai, China) were used. Deionized water was used throughout the experiments. All other chemicals were also of reagent grade.

### 4.2. MDA Treatment

MDA treatment began with 15 g ST (dry weight: 13.734 ± 0.002 g) and 24 mL of the MDA working solution. The mixture was thoroughly stirred to ensure uniform wetting of the biomass, then completely filled into a 150 mL inner bladder of a screw-top pressure-resistant steel reactor and heated in an oven set at 120 °C for 1, 2 and 3 h. When the process was terminated, the products were divided into two parts. One part was dried directly in an oven overnight, coded as PST1, PST2 and PST3 affiliated with its treated time respectively.

The other one was rinsed with water at a solid-to-liquid ratio of 1:15 (g/mL) in a 50 °C water bath for 30 min and then filtered. Repeat the above warm water soaking procedure once. The combined filtrates were collected, and designated as WH1, WH2, and WH3. The solid residues were dried to a constant weight and labeled WST1, WST2, and WST3. For comparison, the untreated ST subjected to the same water-washing and drying procedure was named WST0, and its filtrate was labeled WH0.

### 4.3. AHP Treatment

The solid residues from the previous step were treated with an alkaline hydrogen peroxide (AHP) solution containing 1% (*w*/*v*) NaOH and 1% (*w*/*v*) H_2_O_2_ (hereafter referred to as 1% AHP) at a solid-to-liquid ratio of 1:20 (g/mL) and 90 °C for 1 h. After filtration, the resulting cellulose residues were washed with water to neutrality and dried. These residues were designated as STC0, STC1, STC2, and STC3, corresponding to the initial WST samples.

### 4.4. Compositional Analysis

The cellulose, hemicellulose and lignin contents of all fractionated solid samples (WST0–3, STC0–3) were determined using the Van Soest method [48]. For the water filtrate portions (WH0–3), total sugar, residue sugar and pentosan contents were estimated individually by the phenol-sulfuric acid method [49], the method of Miller using dinitrosalicylic acid [50] and the phloroglucinol method [51]. The yield of solid residual and sugar content of water filtrate were calculated based on the dry weight of ST. The chemical component contents and shift rate were calculated based on the dry weight of its solid residual. The experiments above were conducted in duplicate and the data are expressed as mean ± standard deviation. The shift rate refers to the percentage change in a specific component relative to its original content in the raw material, calculated as [(initial content − final content)/initial content] × 100% for removal rates, or as (final content/initial content) × 100% for retention rates. No inferential statistical tests were performed; therefore, between-treatment differences are described as observed trends and are not interpreted as statistically significant.

### 4.5. Contact Angle Testing

ST, PST1, PST2 and PST3 were accurately weighed (30 mg per sample) and tableted on an infrared tablet press (FW-5A, BOTIAN, Tianjin, China) at a pressure of 15 MPa, lasting 15 s. And then the diffusion and infiltration process of water droplets on the tablets was observed by conducting the contact angle tester (SDS-350, SINDIN, Dongguan, China).

### 4.6. Morphological Analysis Using FE-SEM

A field-emitted scanning electron microscopy (FE-SEM) (SUPRA 55 Sapphire, Carl Zeiss, Oberkochen, Germany) was used to examine the surface morphology of samples and the images were captured at several magnifications. Samples were sputter-coated with gold prior to observation.

### 4.7. X-Ray Diffraction (XRD) Analysis

Crystallinity of samples was analyzed with an X-ray diffractometer (MiniFlex600, Rigaku, Tokyo, Japan) with a range of scattering angle (2*θ*) from 4 to 40° and a scan rate of 2° min^−1^ at 40 kV and 40 mA. The calculation of crystallinity index (*CrI*) was empirical referring to the literature [52]. The crystallinity index (*CrI*) was calculated using the Segal empirical method: *CrI* = [(*I*_200_ − *I*_am_)/*I*_200_] × 100%, where *I*_200_ is the maximum intensity of the (200) lattice diffraction peak at 2*θ* ≈ 22.5°, and *I*_am_ is the intensity of the amorphous background at 2*θ* ≈ 18.5°. XRD measurements were performed in triplicate, and the data are expressed as mean ± standard deviation.

### 4.8. Fourier Transform Infrared (FTIR) Analysis

Infrared spectrogram was employed to identify variations in the functional groups, which was conducted by a FTIR spectrometer (MAGNA-IR 550, Thermo Fisher Scientific, Waltham, MA, USA) in the wavenumber range of 400–4000 cm^−1^ with a resolution of 4 cm^−1^. The quality of each sample was 2 ± 0.1 mg. For hydrogen-bonding analysis, FTIR spectra in the 3100–3700 cm^−1^ region (for ST and PST samples) and the 3000–3800 cm^−1^ region (for STC samples) were subjected to peak deconvolution using OriginPro 2021 (OriginLab Corporation, Northampton, MA, USA) with a Gaussian fitting function. The number of peaks was determined by Origin’s automatic Find Peaks function, and baseline correction was applied using a user-defined baseline with anchor points connected by linear interpolation. The fitting converged with a Chi-Sqr tolerance of 1 × 10^−6^. The deconvoluted peaks were assigned to inter -OH, intra -OH, and free -OH groups based on their peak positions following literature assignments for lignocellulosic hydrogen bonds [16].

### 4.9. Thermogravimetric Analysis (TGA)

Pyrolysis of STC0, STC1, STC2 and STC3 samples were performed via a thermal analyzer (STA449 F3 Jupiter, Netzsch, Selb, Germany), heating from room temperature to 800 °C at a ramp rate of 10 °C·min^−1^ under nitrogen (25 mL·min^−1^) for thermo-gravimetric analysis.

## 5. Conclusions

This study establishes an efficient MDA-AHP strategy for sugarcane tops deconstruction and high-yield recovery of high-purity cellulose. Conducted in a gas–liquid–solid multiphase reaction system at 120 °C, the MDA is proposed to disrupt the lignocellulosic matrix, achieving selective fractionation in which hemicellulose is recovered in aqueous phase, lignin is extracted with 1% AHP, and cellulose is obtained as a solid residue with an 85.33% recovery. A 2 h treatment yields cellulose fibers of 85.82% purity, exposed microfibrillar morphology, high crystallinity (*CrI* = 69.11%), and excellent thermal stability (T_max_ = 344.48 °C), indicating their potential for use in high-value applications such as bio-composites and textiles. Overall, the process avoids hazardous organic solvents and uses comparatively mild treatment conditions, and valorizes agricultural waste, showing potential for sustainable biomass utilization, subject to further process-scale and life-cycle assessments. The specific mechanism of MDA action, including its effect on the degree of polymerization of cellulose, warrants further investigation using advanced analytical techniques. Future work should also focus on the fabrication of cellulose-based composites to evaluate practical performance. Mechanical properties, the degree of polymerization, and end-use performance in composites, films, fibers, textiles, or nanocellulose were not evaluated in this study. Additional limitations include use of a single geographically sourced biomass feedstock and the absence of MDA reuse studies, a complete process mass balance, pilot-scale testing, techno-economic analysis, and quantitative life-cycle and safety assessments.

## Figures and Tables

**Table 1:** The content and FTIR wavenumber assignment of hydrogen bonding in ST and PST samples.

Sample	Inter -OH (%, cm^−1^)				Intra -OH (%, cm^−1^)	Free -OH (%, cm^−1^)	
	Peak 1	Peak 2	Peak 3	Peak 4	Peak 5	Peak 6	Peak 7
ST	0.74 3138	3.43 3178	9.01 3237	30.12 3321	37.97 3443	15.75 3551	2.98 3624
PST1	1.49 3148	9.93 3210	7.00 3267	11.03 3321	53.64 3416	14.78 3544	2.14 3619
PST2	0.83 3142	3.96 3186	18.86 3268	1.28 3343	56.46 3407	18.62 3541	/
PST3	1.23 3146	7.75 3200	5.66 3258	18.39 3314	52.97 3429	14.00 3556	/

**Table 2:** The information on crystallinity, hydrogen bonding and thermal stability of extracted cellulose samples.

Samples	*CrI* (%)	Hydrogen Bonding			TGA/DTG			
		Inter -OH (%)	Intra -OH (%)	Free -OH (%)	R (%) at 150 °C	R (%) at 400 °C	R (%) at 600 °C	T_max_ (°C)
STC0	63.90 ± 0.72	17.60	72.53	9.87	95.19	30.43	21.85	342.23
STC1	65.11 ± 0.81	38.98	55.95	5.07	96.24	25.05	17.96	339.73
STC2	69.11 ± 0.98	53.64	38.82	7.54	96.74	21.91	15.5	344.48
STC3	67.35 ± 0.57	46.65	46.82	6.53	97.15	21.81	15.45	340.74

**Table 3:** Comparative study for cellulose extraction via different processes with the present work.

No.	Ref.	Extraction Process			Solid Yield, %	Chemical Composition, %			Shift Rate, %			Evaluation of Extraction Effect	Evaluation of Extraction Process
		Stages	Parameters	Advantages and Disadvantages		Cellulose	Hemicellulose	Lignin	Cellulose Recovery	Hemicellulose Removal Rate	Lignin Removal Rate		
1	[14]	Raw	Sugarcane trash		100	33.35 ± 0.2	20.26 ± 0.4	22.70 ± 0.8					Environmentally unfriendly, low economic efficiency.
		Organosolv pretreatment	A ternary solvent comprising MIBK, ethanol, and water (25, 42, and 33% *v*/*v*, respectively) with 0.05 M H_2_SO_4_ as a catalyst, 1:10 (*w*/*v*) solid/liquid ratio, stirring at 180 °C for 40 min with an initial pressure of nitrogen at 20 bars.	Liquid-solid reaction, high temperature and complex conditions, producing organic waste and complicating subsequent treatment.	34.7 ± 0.08	69.04 ± 0.1	1.14 ± 0.4	11.01 ± 0.6	71.83	98.05	83.17	Cellulose loss nearly 30%.	
		Bleaching	4% NaOH (1:2 *w*/*v*) and 24% H_2_O_2_ (1:2 *w*/*v*), stirring at 70 °C for 40 min.	Liquid-solid reaction, generating strong alkaline waste and entailing high follow-up treatment intensity.	18.2 ± 0.05	76.22 ± 1.5	0.15 ± 0.02	3.05 ± 0.9	41.60	99.87	97.55	Cellulose loss 58.40%, purity < 80%.	
2	[13]	Raw	Sugarcane straw		100	40.58 ± 0.14	25.77 ± 0.21	31.10 ± 0.92					Traditional process, environmentally unfriendly, low economic efficiency.
		Acid treatment	10% (*v*/*v*) H_2_SO_4_ and 1/10 (*w*/*v*) solid/liquid ratio of dry SCS loading, stirring at 100 °C for 1 h.	Liquid-solid reaction, generating strong acidic waste, entailing high follow-up treatment intensity, and corroding equipment.	60.21	60.29 ± 0.45	12.50 ± 0.58	24.68 ± 0.69	89.45	70.79	52.22	Cellulose loss over 10%.	
		Alkaline treatment	5% (*w*/*v*) NaOH and 1/10 (*w*/*v*) solid/liquid ratio of dry acid pretreated material loading, stirring at 100 °C for 1 h.	Liquid-solid reaction, generating strong alkaline waste and entailing high follow-up treatment intensity.	35.96	83.65 ± 0.56	6.82 ± 0.25	5.78 ± 0.64	74.13	90.48	93.32	Cellulose loss 25.87%, purity < 85%.	
3	[10]	Raw	Sugarcane trash		100	45.2 ± 0.09	23.81 ± 0.85	15.99 ± 0.1					Traditional process, environmentally unfriendly.
		Alkali treated	5% NaOH (*w*/*v*) and 1:25 (*w*/*v*) solid/liquid ratio, stirring at 60 °C for 12 h.	Liquid-solid reaction, featuring long reaction time, generating strong alkaline waste and entailing high follow-up treatment intensity.		63.5 ± 0.15	9.85 ± 0.8	12.95 ± 0.08				Solid mass not provided, cellulose loss unknown.	
		Bleaching	20% H_2_O_2_ and 1:20 (*w*/*v*) solid/liquid ratio, stirring at 80 °C for 8 h.	Liquid-solid reaction, featuring long reaction time, requiring high H_2_O_2_ concentration, and increasing costs.		86.15 ± 0.25	5.2 ± 0.25	5.18 ± 0.05				cellulose loss unknown, purity > 85%.	
4	[12]	Raw	Sugarcane straw: the dried leaves, fresh leaves, and the tops		100	38.1 ± 0.2	29.2 ± 0.3	24.7 ± 0.2					Traditional process, low economic efficiency.
		Steam explosion	A pressure of 15.3 kgf/cm^2^ (equivalent to 200 °C) for 15 min.	Gas–solid reaction, generating no waste liquid, but consuming high energy and demanding stringent equipment requirements.	56.6	50.3 ± 0.5	4.3 ± 0.1	40.4 ± 0.2	74.72	91.67	7.42	Cellulose loss over 25%.	
		Bleaching	1.5 wt.% NaOH and 1:10 (*w*/*v*) solid/liquid ratio, stirring at 98–100 °C for 1 h.	Liquid-solid reaction, generating strong alkaline waste and entailing moderate follow-up treatment intensity.	30.68	77.3 ± 1.2	4.9 ± 0.1	14.8 ± 0.2	62.25	94.85	81.62	Cellulose loss 37.75%, purity < 80%.	
5	[9]	Raw	Sugarcane leaves		100	53.56 ± 0.49	19.90 ± 0.56	20.54 ± 0.26					Traditional process, low economic efficiency.
		Steam explosion	A pressure of 1.3 MPa, 195 °C for 15 min.	Gas–solid reaction, generating no waste liquid, but consuming high energy and demanding stringent equipment requirements.	43.92 ± 4.27	78.44 ± 0.56	1.52 ± 1.21	19.83 ± 0.37	64.32	96.65	57.60	Cellulose loss over 35%.	
		Bleaching	1.4% NaClO_2_ (*w*/*v*) solution and CH_3_COOH to attain pH 4 at 75 °C and the solution was changed every hour until a white sample was obtained.	Multiple liquid-solid reactions, generating large amounts of bleaching waste and increasing the difficulty of follow-up treatment.	23.33 ± 4.11	84.99 ± 0.44	0.94 ± 0.88	4.82 ± 0.98	37.02	98.90	94.53	Cellulose loss 62.98%, purity approx 85%.	
6	Present study	Raw	Sugarcane tops: the apical growing stem portion with tender leaves		100	40.19 ± 0.13	37.75 ± 0.07	7.95 ± 0.13					Eco-friendly and energy-saving, with high potential economic benefits.
		MDA treatment	MDA solution and 1:1.6 (*w*/*v*) solid/liquid ratio, stirred until uniformly wetted, heated at 120 °C for 2h.	Gas–liquid–solid multiphase reaction, producing no waste liquid and requiring mild reaction conditions.	60.26 ± 0.04	62.99 ± 0.60	11.74 ± 0.40	10.74 ± 0.07	94.39 ± 0.94	81.26 ± 0.62	18.61 ± 0.48	Cellulose loss only 5.61%.	
		AHP treatment	1% NaOH (*w*/*v*) and 1% H_2_O_2_ (*w*/*v*), and 1:20 (*w*/*v*) solid/liquid ratio, stirring at 90 °C for 1 h.	Liquid-solid reaction, featuring relatively low concentrations of NaOH and H_2_O_2_, and greatly reducing follow-up treatment difficulty.	39.67 ± 0.26	85.82 ± 0.20	7.65 ± 0.09	3.01 ± 0.03	85.33 ± 0.63	91.90 ± 0.39	84.89 ± 0.39	Cellulose loss 14.67%, purity > 85%	

## Data Availability

The original contributions presented in this study are included in the article/Supplementary Material. Further inquiries can be directed to the corresponding author.

## Supplementary Materials

The following supporting information can be downloaded at https://www.mdpi.com/article/10.3390/molecules31162860/s1, Figure S1: Microscopic observations of lignin-stained samples via the modified Wiesner reaction: (a,b) ST at 10×/100× mag., respectively; (c,d) PST2 at 10×/100× mag., respectively; Figure S2. SEM of the epidermal tissues of WST0 (a: 100X; b: 500X) and WST2 (c: 100X; d: 500X); Figure S3. SEM of the conduction tissues of WST0 (a: 500X; b: 1000X; c: 10,000X) and WST2 (d: 500X; e: 1000X; f: 10,000X); Figure S4. The peak separation of hydrogen bonding OH stretching from 3000 to 3800 cm^−1^ of STC0 (a), STC1 (b), STC2 (c) and STC3 (d); Table S1. Information of inter -OH (peak 1 and 2), intra -OH (peak 3 and 4) and free -OH (peak 5 and 6) from Figure S4; Table S2. Summary of FTIR peak deconvolution parameters for PST and STC samples.
