# Supplementary material for: Two-Step Cascade Process for Efficient Isolation of Cellulose from Sugarcane Trash
Source: Molecules. 2026 Aug 16;31(16):2860. doi: 10.3390/molecules31162860 (PMC13515468; doi:10.3390/molecules31162860)
Supplement: Supplementary file 1 [file molecules-31-02860-s001.zip › molecules-4419011-supplementary.pdf]

Supporting Information

Title

Two-step Cascade Process for Efficient Isolation of Cellulose from Sugarcane Trash

Authors

Jian Zhang [1,2,†], Jiang-Lin Yin [2,†], Liu-Yi Chen [1], Jie Gao [1], Bao-Yu Huang [1], Li-Ting Gao [1], Mei-Xia He [1], Xiang-Yi Li [1], Ri-Hui Lin [1,*] and Da-Wei Li [3,*]

Affiliations

[1]School of Chemistry and Chemical Engineering, Guangxi Key Laboratory for Polysaccharide Materials and Modification, Guangxi Minzu University, Nanning 530006, Guangxi, China

[2]Institute of Traditional Chinese and Zhuang-Yao Ethnic Medicine, Guangxi University of Chinese Medicine, Nanning 530200, China

[3]Key Laboratory of Eutrophication and Red Tide Prevention of Guangdong Higher Education Institutes, College of Life Science and Technology, Jinan University, Guangzhou 510632, China

[*]Corresponding author. Email: rihuilin@gxmzu.edu.cn , daweili@jnu.edu.cn

[†]These authors contributed equally to this work

Summary: 7 pages, 4 figures, 2 tables.

The number of pages:7

Figure S1. Microscopic observations of lignin-stained samples via the modified Wiesner reaction: (a, b) ST at 10×/100× mag., respectively; (c, d) PST2 at 10×/100× mag., respectively.

Figure S2. SEM of the epidermal tissues of WST0 (a: 100X; b: 500X) and WST2 (c: 100X; d: 500X)

Figure S3. SEM of the conduction tissues of WST0 (a: 500X; b: 1000X; c:10,000X) and WST2 (d: 500X; e: 1000X; f:10,000X)

Figure S4. The peak separation of hydrogen bonding OH stretching from 3000 to 3800 cm-1 of STC0 (a), STC1 (b), STC2 (c) and STC3 (d)

Table S1. Information of inter -OH (peak 1 and 2), intra -OH (peak 3 and 4) and free -OH (peak 5 and 6) from Figure S4

Table S2. Summary of FTIR peak deconvolution parameters for PST and STC samples in the 3100–3700 cm^{-1} region

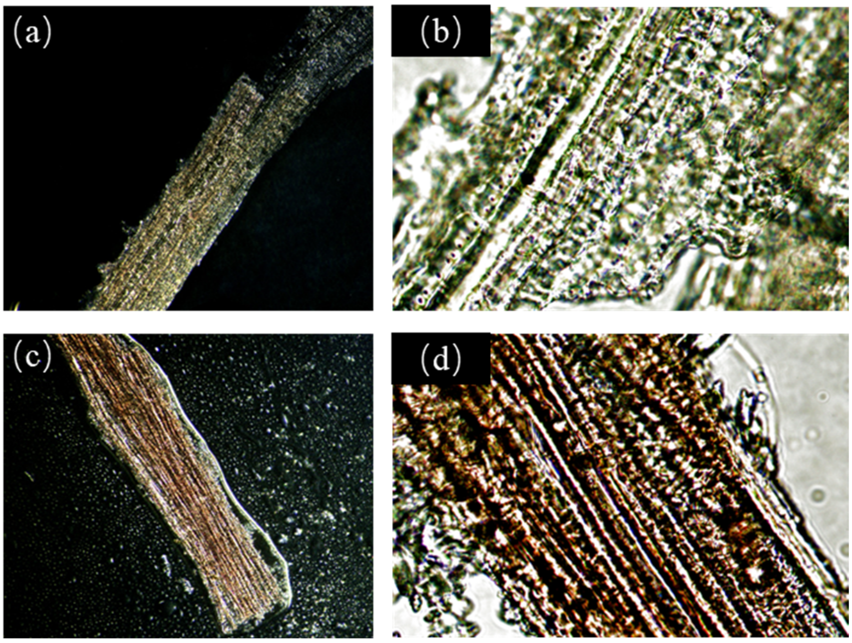

Figure S1. Microscopic observations of lignin-stained samples via the modified Wiesner reaction: (a, b) ST at 10×/100× mag., respectively; (c, d) PST2 at 10×/100× mag., respectively.

The procedure of Wiesner reaction with slightly modification was as followed: small pieces of the sample were placed on a clean glass slide, and a sufficient volume of 2% phloroglucinol (dissolved in 95% ethanol) was added dropwise to cover the sample completely. After incubation at room temperature for 5 min, an equal volume of 12% hydrochloric acid was added (to maintain the optimal reaction ratio), and the mixture was allowed to stand for 1 min to facilitate sufficient color development. Finally, the staining of the sample was observed under visible light using an optical microscope, and micrographs of the target areas were captured for documentation.

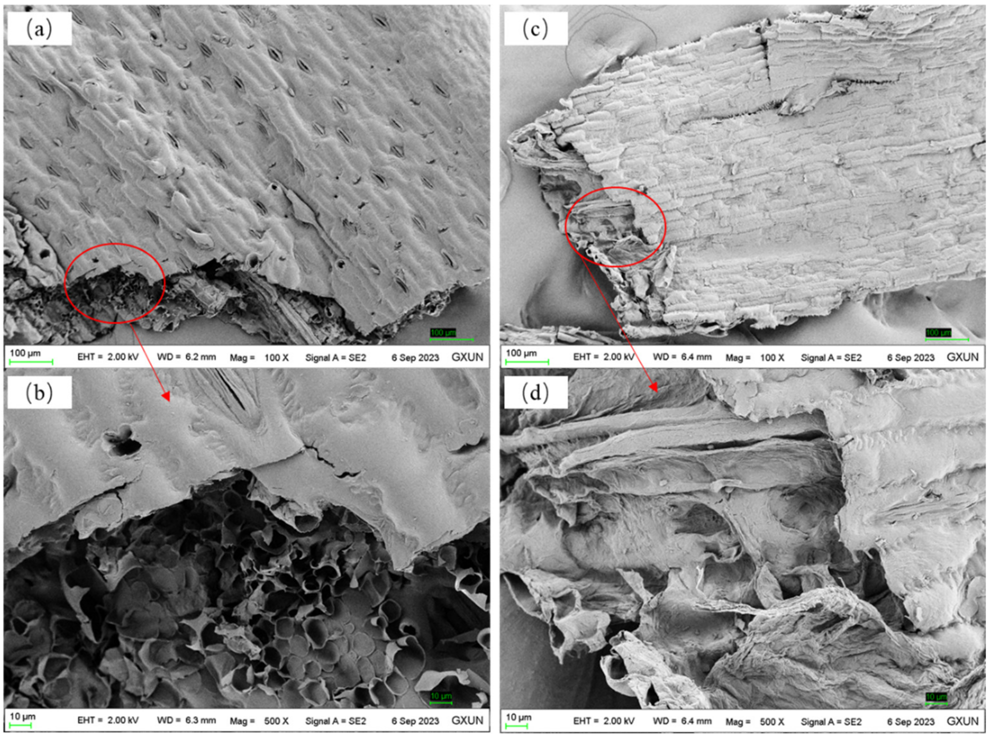

Figure S2. SEM of the epidermal tissues of WST0 (a: 100X; b: 500X) and WST2 (c: 100X; d: 500X)

Figure S2 shows that the epidermal tissue of WST0 exhibits a regular lamellar texture at both 100X and 500X magnifications, with uniformly arranged and fine structures. In contrast, the epidermal tissue of WST2 appears rough and fluffy with numerous damages and folds even at 100X; at 500X, the lamellar structure becomes more fragmented and irregular.

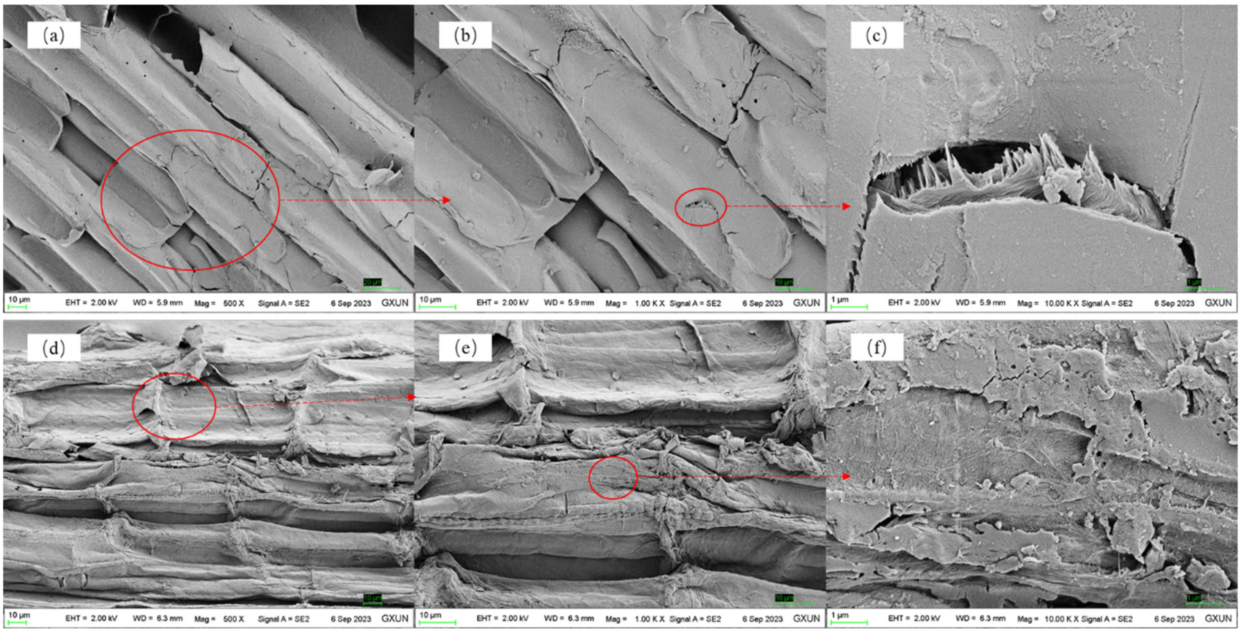

Figure S3. SEM of the conduction tissues of WST0 (a: 500X; b: 1000X; c:10000X) and WST2 (d: 500X; e: 1000X; f:10000X)

As seen in Figure S3, the conduction tissue of WST0 presents a continuous and neat tubular/lamellar structure from 500X to 10000X magnifications, featuring good integrity and clear micro-texture. In contrast, the conduction tissue of WST2 is loose and discontinuous at all magnifications: obvious fractures and folds can be seen at 500X and 1000X; at 10000X, interlayer fragmentation is prominent with local exposure of microfibrils, creating a sharp contrast with the regularity of WST0.

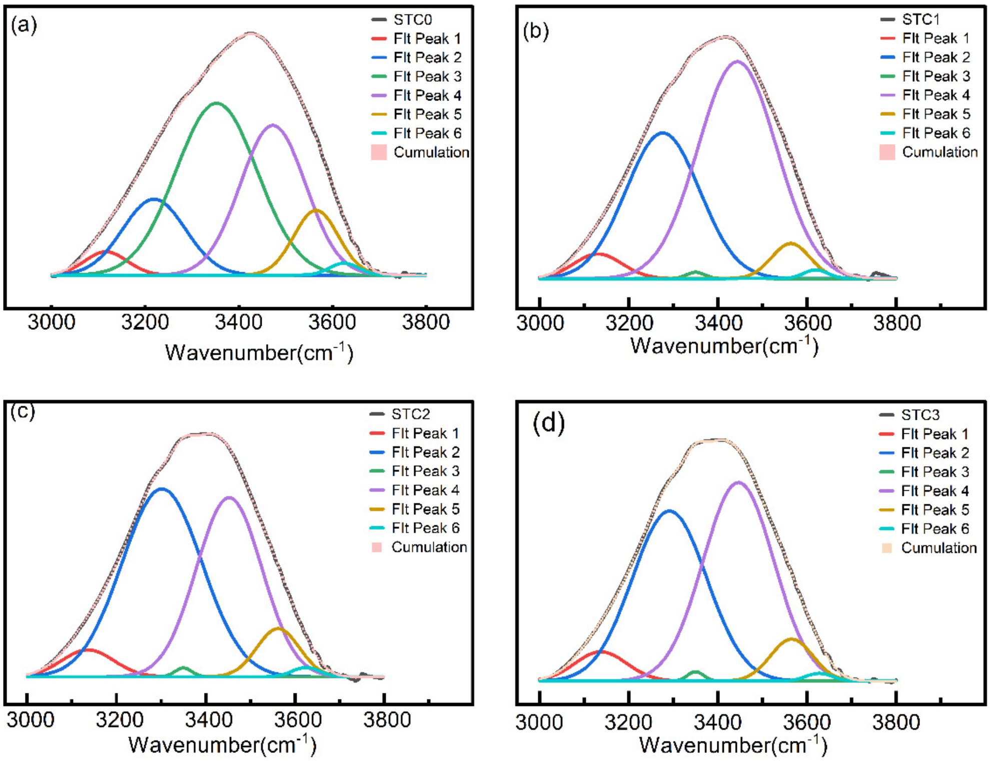

Figure S4. The peak separation of hydrogen bonding OH stretching from 3000 to 3800 cm^{-1} of STC0 (a), STC1 (b), STC2 (c) and STC3 (d)

Table S1. Information of inter -OH (peak 1 and 2), intra -OH (peak 3 and 4) and free -OH (peak 5 and 6) from Figure S4

Sample	Index	Inter -OH		Intra -OH		Free -OH	
		Peak 1	Peak 2	Peak 3	Peak 4	Peak 5	Peak 6
STC0	Percentage (%)	3.03	14.57	42.65	29.89	8.80	1.07
	Wavenumber (cm^{-1})	3116	3219	3352	3473	3565	3625
STC1	Percentage (%)	3.84	35.14	0.37	55.58	4.39	0.68
	Wavenumber (cm^{-1})	3130	3276	3350	3443	3564	3620
STC2	Percentage (%)	4.55	49.09	0.49	38.33	6.69	0.86
	Wavenumber (cm^{-1})	3136	3302	3350	3453	3562	3625
STC3	Percentage (%)	4.96	41.69	0.51	46.31	5.83	0.70
	Wavenumber (cm^{-1})	3137	3292	3350	3446	3565	3627

Table S2. Summary of FTIR peak deconvolution parameters for PST and STC samples

Sample	Number of Peaks	R^2 (COD)	Reduced Chi-Sqr	Baseline Anchors
ST	7	0.99977	5.06×10^{-8}	2
PST1	7	0.99987	8.21×10^{-8}	2
PST2	6	0.99987	1.75×10^{-7}	2
PST3	6	0.99976	2.17×10^{-7}	2
STC0	6	0.99988	4.90×10^{-7}	4
STC1	6	0.99996	3.57×10^{-7}	4
STC2	6	0.99988	8.29×10^{-7}	4
STC3	8	0.99992	7.52×10^{-7}	4
